# Metastatic Castration-Resistant Prostate Cancer: Advances in Treatment and Symptom Management

**DOI:** 10.1007/s11864-024-01215-2

**Published:** 2024-06-24

**Authors:** Tivya Kulasegaran, Niara Oliveira

**Affiliations:** 1 Mater Hospital Brisbane, Cancer Centre, Raymond Terrace, South Brisbane, QLD 4104 Australia; 2 School of Clinical Medicine, Mater Clinical Unit, The University of Queensland, Brisbane, Queensland, Australia, Raymond Terrace, South Brisbane, QLD 4101 Australia

**Keywords:** Prostate cancer, Castration resistance, Precision oncology, New therapies, Targeted therapies, Cancer symptoms

## Abstract

The management of metastatic castrate-resistant prostate cancer (mCRPC) has evolved in the past decade due to substantial advances in understanding the genomic landscape and biology underpinning this form of prostate cancer. The implementation of various therapeutic agents has improved overall survival but despite the promising advances in therapeutic options, mCRPC remains incurable. The focus of treatment should be not only to improve survival but also to preserve the patient’s quality of life (QoL) and ameliorate cancer-related symptoms such as pain. The choice and sequence of therapy for mCRPC patients are complex and influenced by various factors, such as side effects, disease burden, treatment history, comorbidities, patient preference and, more recently, the presence of actionable genomic alterations or biomarkers. Docetaxel is the first-line treatment for chemo-naïve patients with good performance status and those who have yet to progress on docetaxel in the castration-sensitive setting. Novel androgen agents (NHAs), such as abiraterone and enzalutamide, are effective treatment options that are utilized as second-line options. These medications can be considered upfront in frail patients or patients who are NHA naïve. Current guidelines recommend genetic testing in mCRPC for mutations in DNA repair deficiency genes to inform treatment decisions, as for example in breast cancer gene mutation testing. Other potential biomarkers being investigated include phosphatase and tensin homologues and homologous recombination repair genes. Despite a growing number of studies incorporating biomarkers in their trial designs, to date, only olaparib in the PROFOUND study and lutetium-177 in the VISION trial have improved survival. This is an unmet need, and future trials should focus on biomarker-guided treatment strategies. The advent of novel noncytotoxic agents has enhanced targeted drug delivery and improved treatment responses with favourable toxicity profiling. Trials should continue to incorporate and report health-related QoL scores and functional assessments into their trial designs.

## Introduction

Prostate cancer is the second most frequent cause of cancer among males and caused an estimated 385,000 deaths worldwide in 2018. With the ageing population, the rates of new prostate cancer (PC) diagnoses and mortality are expected to increase [1]. It is estimated that 65% of all new PC diagnoses are made in men aged above 65 years, and 25% of all new PC diagnoses are made in men aged over 75 years [2].

Metastatic PC generally responds to initial androgen deprivation therapy. However, most patients will inevitably develop treatment resistance and transition to a more aggressive disease phenotype. This progression of the disease, regardless of castrate testosterone levels, is termed ‘castrate-resistant’ and is often more refractory to systemic treatment [3, 4].

Despite significant advancements in therapies, metastatic castrate-resistant PC (mCRPC) remains incurable and is capable of causing considerable disease burden. It is a heterogeneous disease that is associated with high mortality and morbidity [5, 6]. The majority of men who are diagnosed with localized mCRPC develop distant metastases [7].

The natural progression of mCRPC often involves worsening symptoms, such as fatigue and bone pain, which can be detrimental to the patient’s overall quality of life (QoL). The goal of treatment is thus not only to improve survival but also to preserve patients’ QoL and alleviate cancer-related pain and symptoms [8, 9].

Chemotherapy treatments such as docetaxel remain the recommended first-line therapy for patients with good performance status and in those with no previous progression on docetaxel. Novel hormonal agent (NHA) therapy is generally reserved for second-line treatment after progression on chemotherapy or for patients who are deemed unfit or wish to avoid cytotoxic chemotherapy [5, 10].

The identification of specific gene aberrations and mutations with actionable potential has made precision medicine a valuable tool for guiding treatment decisions. Examples include poly (ADP-ribose) polymerase inhibitors (PARPis) for homologous recombination repair (HRR) loss and protein kinase B (AKT) pathway inhibitors for treating tumours with phosphatase and tensin homologue (PTEN) loss [11, 12••].

Herein, we discuss the most recent advancements in mCRPC treatment, the mechanisms of action, the nuances of trials, and the direction in which this field is heading. We also summarize ongoing, in-development treatment options and the challenges of integrating these agents into clinical practice.

## Chemotherapy

The use of docetaxel has been the standard chemotherapy approach for more than two decades. In a phase III trial of more than 1006 patients (TAX 327), the participants were randomized into either a docetaxel and prednisone arm at two doses (three times weekly at 75 mg/m^2^ or weekly at 30 mg/m^2^) or the mitoxantrone and prednisone arm (control). The trial met its primary endpoint, with a median overall survival (OS) of 19.2 months for patients receiving three weekly doses of docetaxel compared with 16.3 months for patients in the control arm (*p* < 0.004). The OS benefit in the weekly docetaxel regimen was non-significantly superior to the mitoxantrone arm. Common toxicities included diarrhoea (32%) and neuropathy (30%). The incidence of grade 3/4 neutropenia was 32%, with 26% of patients experiencing at least one severe adverse event [5, 10, 13].

Carbazitaxel is another taxane chemotherapy that is used in the docetaxel-resistant setting. The pivotal TROPIC (2010) trial randomized 775 patients with mCRPC who had received prior treatment with docetaxel to either cabazitaxel and prednisone or mitoxantrone and prednisolone. The trial was positive, with a significantly longer OS in the experimental arm (15.1 vs 12.7 months), a hazard ratio (HR) of 0.7, *p* ≤ 0.0001. Secondary outcomes favoured docetaxel with better PSA response, reduction in pain and more prolonged progression-free survival (PFS). These results were statistically significant [14].

The toxicities associated with carbazitaxel are predominantly haematologic, with any-grade neutropenia accounting for 94% of patients with grade 3/4 neutropenia (82%) and febrile neutropenia (8%). Other toxicities included grade 3–4 diarrhoea (6%), grade 3–4 nausea (2%) and grade 3–4 fatigue (5%) [14].

## Androgen receptor signalling inhibitors

Novel anti-androgen agents have demonstrated clinical benefits in both chemotherapy-naïve and second-line treatment settings and are currently approved therapeutic agents [5, 10]. The androgen signalling pathway plays a pivotal role in the development of castration resistance. Several abnormal aberrations in the androgen receptor (AR) axis, such as gene amplification and overexpression, contribute to castration resistance. Androgen receptor signalling inhibitors (ARSIs) work by antagonizing AR function, resulting in the blockade of androgen production and a further reduction in testosterone production [15••].

### Enzalutamide

Enzalutamide is a potent nonsteroidal AR inhibitor that binds to AR with high affinity, antagonizes testosterone binding to AR and impairs nuclear localisation and transcriptional activity [5, 16]. The AFFIRM (2012) and PREVAIL (2014) trials were pivotal in terms of demonstrating efficacy in mCRPC in pre- and postdocetaxel settings. The AFFIRM trial included patients who had already received docetaxel and demonstrated an improvement in OS time in the treatment arm (18.4 vs 13.6 months, HR, 0.63). Secondary outcome measures, such as the PSA response rate, time to PSA progression and radiographic PFS, were superior in the treatment arm. Enzalutamide was associated with increased neurotoxicity (convulsions) in less than 1% of patients. Other notable toxicities observed in the experimental arm included arterial hypertension (6% vs 3%), asthenia (34% vs 29%) and hot flushes (20% vs 10%) [5, 17].

The PREVAIL trial examined enzalutamide administered predocetaxel vs a placebo. This was a positive trial, with patients in the enzalutamide arm achieving better radiographic PFS (rPFS) at 12 months (65% vs 14%, HR 0.19, *p* < 0.001). The OS time was also significantly longer in the experimental arm (32.4 vs 30.2 months, HR 0.7, *p* < 0.001) [5, 21].

The results from these two trials led to the approval of enzalutamide for use in pre- and postdocetaxel settings [5].

### Abiraterone

Abiraterone is a specific cytochrome 17α-hydroxylase inhibitor that is critical for androgen synthesis. It also inhibits the enzymes 3β-hydroxysteroid dehydrogenase, steroid 11β-hydroxylase and steroid 21-hydroxylase, which can result in increased mineralocorticoid production. This can lead to toxicity, such as hypokalaemia, fluid retention and adverse cardiovascular reactions [22, 23].

COU-AA-30123 and COU-AA 30224 were two trials that demonstrated a survival benefit for abiraterone in both docetaxel pretreatment (median OS: 14.8 vs 10.9 months, HR 0.65, 95% confidence interval [CI], 0.54–0.77) and posttreatment (median OS: 34.7 vs 30.3 months, HR 0.81, 95% CI, 0.70–0.93) settings. Abiraterone was also superior in halving of PSA and in prolonging PFS.

Treatment-related toxicity, including arterial hypertension, hypokalaemia and peripheral oedema, occurs mainly due to an excess of mineralocorticoids. This effect can be mitigated by the concurrent use of low-dose prednisolone [24, 25].

## Targeted molecular therapies

The genomic profiling of tumours is a fundamental component of precision oncology. It has emerged as an invaluable tool that can provide insight into tumour biology and guide treatment decisions. Identifying actionable genomic mutations and pathways allows for the implementation of additional therapeutic agents. Generally, whole-genome testing is conducted on a tissue biopsy. However, innovative technology using liquid biopsy or circulating tumour DNA (ctDNA) has also garnered increasing interest, with several clinical trials currently investigating its use in clinical practice [26]. The genomic landscape of PC is dynamic and evolves due to various factors, such as treatment pressure and the emergence of resistant ctDNA clones. Minimally invasive liquid biopsy procedures have emerged as valuable tools for detecting heterogeneity and tracking acquired mutations in real time [27, 28].

A complex and heterogeneous disease, mCRPC is often characterized by high levels of genomic and molecular alterations. One study reported that 40%–60% of mCRPC cases exhibit aberrations in the AR, erythroblast transformation specific, tumour protein p53 and PTEN genes, with 20%–25% harbouring somatic or germline alternations in DNA repair genes involved in homologous recombination [29].

### Deoxyribonucleic (DNA) repair pathway

Approximately 20% of mCRPC patients harbour abnormalities that affect DNA repair genes [12••]. This process is called homologous recombination repair (HRR), which accurately repairs double-strand breaks during the synthesis (S) and Gap 2 (G2) phases of the cell cycle [30]. Tumour cells with a deficiency in the HRR pathway are, therefore, unable to accurately repair double-strand breaks, which results in high failure rates. Common genetic mutations affecting the HRR include those in the breast cancer 2 (BRCA2), CHEK2, ATM and BRCA1 genes. These gene alterations can occur at either the acquired somatic or germline level [7, 12••].

Deleterious gene alterations related to HRRs, such as those in BRCA1/2, sensitizes prostate cancer cells to PARP inhibition. PARP is a protein which repairs single-strand breaks. PARP inhibition leads to unrepaired single-strand DNA breaks, resulting in an excess of double-stranded DNA which promotes cell death. Cells with HRR dysfunction are unable to repair themselves, resulting in cell cycle arrest and ultimately cell apoptosis. On the other hand, HRR-proficient cells with functional PARP can self-repair and survive.

Synthetic lethality refers to when both a PARPi and HRR dysfunction is required for cell cycle arrest. As the presence of only one deficient pathway will not independently cause cell death. This explains the highly selective targeting of tumour cells by PARPi [31].

#### PARPi monotherapy

Over the past several years, multiple clinical trials have demonstrated the efficacy of PARP as monotherapy for HRR-deficient mCRPC in a second-line setting following ARSIs. The results of this research have led to its approval for use in patients with an HRR deficiency [5, 32, 33].

The phase III PROfound study examined the use of olaparib in 387 patients with mCRPC progressing after docetaxel and an ARSI. Tissue samples of the participants were tested to determine their HRR status. This was a biomarker guided trial, patients with at least one alteration in BRCA1, BRCA2 or ATM were allocated into cohort A. Patients with other HRR genomic alternations such as PALB2, RAD51B and RAD 51C mutation were allocated into cohort B. Patients either had Olaparib or the alternate ARSI (commonly referred as ARSI-switch).

This was a positive trial, in cohort A, there was an improvement in radiological PFS in the olaparib arm compared to the control arm (7.4 vs 3.6 months, HR 0.34, *p* < 0.001). The objective response rate (ORR) was 33% in the olaparib arm and 2% in the control arm. The median overall survival in cohort A was 18.5 months in the olaparib arm and 15.1 months in the control arm, this was despite an 81% crossover from the control arm to the experimental arm on progression. Notable toxicities included reversible anaemia and a small but significant risk for acute myeloid leukaemia and myelodysplasia (MDS), which was less than 1%. Other ongoing PARPi monotherapy trials are outlined in Table 1 [18, 34].

**Table 1:** Summary of poly (ADP-ribose) polymerase (PARP) inhibitor (PARPi) monotherapy trials for metastatic castrate-resistant prostate cancer

Study	Phase of trial (Number of patients)	Treatment arm	Control arm	Patient population	Biomarker cohort	Results Primary and secondary endpoint
PROFOUND [18]	Phase III (387)	Olaparib 300 mg BD	Enzalutamide 160 mg OD or Abiraterone 100 mg od	mCRPC (patients have progressed on ARSI)	Cohort A: BRCA 1 and 2, ATM Cohort B: Other HRR +	**Primary endpoint**: rPFS 5.8 (olaparib) vs 3.5 months (ARSI) rPFS (BRCA 1/2, ATM) 7.4 (olaparib) vs 3.6 months (ARSI) HR 0.34 **Secondary endpoint:** Median OS 18.5 months in the olaparib group vs 15.1 months in the control group ORR: 33% vs 2%
GALAHAD [19]	Phase II Single arm (289)	Niraparib 300 mg OD	–	mCRPC (patients have progressed on taxane and ARSI)	Biallelic BRCA 1/2 mutation	**Primary endpoint:** ORR 41% **Secondary endpoint:** Median rPFS 8.2 months
TALAPRO-1 [20]	Phase II Single-arm	Talazoparib 1 mg OD	–	mCRPC (patients have progressed on taxane and ARSI)	HRR+	**Primary endpoint:** ORR 29.8% ORR: BRCA1/2 46% **Secondary endpoint:** Median rPFS 8.2 months

Abbreviations: *OD* once daily, *BD* twice daily, *mCRPC* metastatic castrate-resistant prostate cancer, *rPFS* radiological progression-free survival, *ARSI* androgen receptor signalling inhibitor, *mOS* median overall survival, *ORR* objective response rate, *HRR+* homologous repair recombination mutant

Emerging data from several trials have reported that ARSIs (either enzalutamide or abiraterone) combined with a PARPi have a PFS advantage relative to enzalutamide or abiraterone alone. The rationale for this combination is to exploit the potential synergy when co-targeting the AR and DNA repair mechanisms to slow tumour proliferation. Furthermore, PARPi upregulate AR signalling, thereby enhancing ARSI activity. Furthermore, ARSIs block the transcription of some HRR genes, inducing an HRR deficiency-like state and sensitizing cells to PARPi activity [35, 36].

#### Combining PARPis and ARSIs

The PROpel, MAGNITUDE and TALAPRO-2 trials examined the combination of ARSIs and PARPis in the first-line setting for mCRPC [35–37].

The phase III PROpel study explored the use of abiraterone plus olaparib vs abiraterone plus placebo in patients with mCRPC in a first-line setting. Patients were recruited regardless of their HRR status. The intention-to-treat (ITT) analysis of PFS was 24.8 months in the abiraterone and olaparib combination arm and 16.6 months in the abiraterone plus placebo arm (HR 0.66, *p* < 0.001). The median OS improved in the treatment arm at 42.1 vs 34.7 months, favouring the olaparib and abiraterone combination. A trend toward improved OS was observed in all subgroups, including the HRR mutant, non-HRR mutant, BRCA mutation and non-BRCA mutation subgroups. The most significant improvement was again observed in the BRCA mutation subgroup (HR 0.29, 95% CI, 0.14–0.56). This trial concluded that abiraterone and olaparib statistically and clinically improved the rPFS, regardless of the HRR status [37].

The toxicity profiles were consistent with the existing data, with a greater proportion of patients receiving abiraterone plus olaparib having grade 3 or higher adverse events than those receiving abiraterone alone (47% vs 38%). The most common adverse event with olaparib was anaemia, which occurred in 46% of patients. Severe-grade 3–4 anaemia was reported in 15.1% of the patients. The incidence of MDS was not recorded [5, 37].

The phase III MAGNITUDE trial assessed the efficacy and safety profile of niraparib with abiraterone acetate and prednisone (AAP) in patients with mCRPC. Patients were stratified according to the presence or absence of HRR biomarker positivity [38]. The trial noted that the combination of niraparib and AAP improved the primary endpoint of rPFS in patients with BRCA1/2 mutations and in the overall HRR+ cohort. The median rPFS was longer in the BRCA1/2 mutation cohort, with an HR of 0.53. However, in the HRR-negative group, the results were negative. Overall, the combination was well tolerated, with grade 3 anaemia accounting for 28.3% and hypertension accounting for 14.6% of all toxicity [38].

The TALAPRO-2 trial examined the use of talazoparib, a novel PARPi, with the addition of enzalutamide in a first-line setting for the treatment of mCRPC. This was another biomarker-guided trial in which patients were separated according to their HRR status. The combination resulted in clinically meaningful and statistically significant improvements in the rPFS. At the time of the preliminary analysis, the median rPFS had not yet been reached for the combination group vs. the placebo plus enzalutamide group after 27.5 months. This represents a 37% improvement in the median PFS length based on imaging. A post hoc analysis revealed that the greatest benefit was observed in those who harboured BRCA1/2 mutations, with a hazard ratio of 0.2. In the HRR-deficient group, the HR was 0.46. Interestingly, a benefit was also observed in the unselected gene alternation group, with an HR of 0.66, exceeding the prespecified HR of 0.696 for significance [39].

The above trials support the use of PARPis as biomarkers in mCRPC treatment and support the use of the HRR status as a promising biomarker for selecting patient cohorts that may benefit from the addition of PARPis. These results are summarized in Table 2.

**Table 2:** Summary of poly (ADP-ribose) polymerase (PARP) inhibitor (PARPi) and androgen receptor signalling inhibitor (ARSI) trials in metastatic castration-resistant prostate cancer (mCRPC)

Study	Phase of trial (Number of patients)	Treatment arm	Control arm	Patient population	Biomarker cohort	Results Primary and secondary endpoint	ORR (%)
PROpel [37]	Phase III (796)	Olaparib 300 mg BD/Abiraterone 1000 mg OD	Placebo/Abiraterone 1000 mg OD	First-line mCRPC (prior docetaxel in the metastatic castrate-sensitive setting allowed, NHA, PARPi not allowed)	All comers ctDNA-based testing conducted retrospectively	**Primary endpoint:** rPFS 24.8 (olaparib/abiraterone) vs 16.6 months (placebo/olaparib) (HR 0.66) HHRm cohort: median rPFS 28.8 (olaparib/abiraterone) vs 13.8 months (HR: 0.45) Non-HRRm cohort: Median rPFS: 27.6 (olaparib/abiraterone) vs 19.1 months (HR 0.72) BRCAm cohort: Median rPFS NR olaparib/abiraterone vs 8.4 months placebo/abiraterone (HR 0.18) **Secondary endpoint:** OS in ITT population, median OS 42.1 vs 34.7 months	58.4% vs 48.1%
MAGNITUDE [38]	Phase III (670)	Niraparib 200 mg OD/Abiraterone 1000 mg OD	Placebo	First-line mCRPC (less than 4 months prior abiraterone therapy was allowed)	Cohort A (without gene alternations) Cohort B with HRR gene alternations	**Primary endpoint** rPFS BRCA1/2 alternations 16.6 vs 10.9 months (HR 0.53) rPFS in HRR + ve 16.5 vs 13.7 months Prespecified futility analysis showed no benefit in HRR negative cohort **Secondary endpoint:** OS still immature	60% vs 28%
TALAPRO-2 [39]	Phase III (1095)	Talazoparib/enzalutamide	Placebo/enzalutamide	First line mCRPC, first line ARPi allowed in castrate- sensitive setting	Cohort 1: All comers, HRR unselected Cohort 2: Patients with HRR gene alterations	**Primary endpoint:** rPFS (in ITT population) NR vs 27.5 months (HR: 0.63)	61.7% vs 43.9%

Abbreviations: *OD* once daily, *BD* twice daily, *mCRPC* metastatic castration-resistant prostate cancer, *rPFS* radiological progression-free survival, *ARSI* androgen receptor signalling inhibitor, *mOS2* median overall survival, *ORR* objective response rate, *HRR+* homologous repair recombination mutant

## The PTEN/AKT pathway

Dysregulation of the phosphatidylinositol 3-kinase (PI3K)/AKT (AKT serine/threonine kinase)/mammalian target of rapamycin (mTOR) signalling pathway is linked to the progression of prostate cancer. It is an important pathway for cell growth, survival and proliferation. This dysregulation is generally caused by the loss of the PTEN tumour suppressor gene, which negatively regulates this pathway [40]. It is estimated that 10%–15% of all primary prostate tumours harbour this mutation, with a higher prevalence (40%–60%) in the castrate resistant metastatic setting [41]. Loss of PTEN leads to hyperactivation of the PI3K/AKT/mTOR pathway, which is associated with poor clinical outcomes and treatment resistance. Therefore, PTEN deficiency is a potential predictive biomarker because it confers resistance to anti-androgen therapy. However, targeting this pathway in prostate cancer cells is limited by significant cross-talk. Since PTEN is integral to multiple cellular pathways, inhibiting this may affect non-cancerous cells and lead to toxicities at therapeutic doses [42]. This interaction is referred to as cross talk.

There is growing interest in exploring novel therapeutic agents to directly restore PTEN in deficient prostate tumours and inhibit the PI3K/AKT/mTOR pathway [39].

Capivasertib is a selective pan-AKT inhibitor that targets all three AKT isoforms (AKT1/2/3). The ProCAID trial is a phase II trial that investigated the use of capivasertib in combination with docetaxel [43]. The trial did not meet its composite primary endpoint which included PFS and PSA reduction. However, it met its secondary endpoint of OS. Further subgroup analysis at maturity revealed that this improvement in OS was only observed in patients who progressed on ARSIs [40]. Despite this being a negative trial, the improvement in OS was intriguing and hypothesis generating. These results lead to the phase III trial, CAPItello 280.

CAPItello-280 is under recruitment and will examine capivasertib in combination with docetaxel compared with placebo and docetaxel in patients whose disease has progressed on ARSIs in any setting. The primary endpoint will be OS [44]. The results from this trial will provide insight into the selection of potential candidates for this therapeutic strategy.

Ipatasertib is another novel selective inhibitor of all three AKT isoforms. The IPATential 150 double-blind phase III trial randomized ipatasertib and abiraterone/prednisolone vs abiraterone/prednisolone alone. The rationale for this approach was that a dual-activation pathway would enhance synergistic antitumour activity for a better clinical outcome. The study was positive in that patients who had PTEN loss on immunohistochemistry had better rPFS (HR 0.65, 95% CI, 0.45–0.95, *p =* 0.0206) with the combination of ipatasertib and abiraterone [45].

While AKT inhibitors potentially represent a viable therapeutic strategy for treating mCRPC, the challenge remains to better target AKT isomers. With newer trials examining their use in hormone-sensitive settings, the challenge remains regarding how to best optimize study designs and endpoints, as well as understanding their unique and complex biology and their efficacy before their incorporation into standard practice [40, 46].

## Immunotherapy

Although immunotherapy has revolutionized the treatment of many solid organ tumours, it has only recently been studied in advanced prostate cancer.

Although initial preclinical studies showed promise, these have not translated into positive outcomes in later-stage clinical trials, with trials being curtailed due to futility [47–49].

The identification of potential biomarkers to help guide treatment decisions in this space is conflicting and limited. *Programmed death-ligand 1* (PD-L1) positivity did not correlate with disease response in the Keynote-199 trial, although the low patient sample size was small, with only 5% of patients expressing PD-L1 [50]. Mismatched repair deficiency (dMMR) and a high tumours mutational burden (TMB) greater than 10 mut/Mb generally confer sensitivity to immune checkpoint inhibitors. Typically, a more aggressive disease, dMMR PC, has a poorer response to conventional treatment and accounts for only 1–5% of all prostate cancers. The cut-off and validation of TMB status assessments remain uncertain, as data are often extrapolated from other tumours streams [51].

When used as monotherapy or in combination with chemotherapy, NHA and PARPi, patients with biomarker-unselected advanced PC have not benefited from immunotherapy. Additionally, PD-L1 expression has not been proven to be a reliable predictive biomarker of response to pembrolizumab. This reflects an immunologically cold tumour microenvironment, which is unfavourable for checkpoint inhibitors to act upon in prostate cancer. Future immunotherapy trials should emphasize better patient selection and biomarker-guided treatment strategies [5, 51].

## Theranostics

Theranostics is an emerging field of medicine that combines diagnostic imaging and treatment by delivery of targeted radioactive isotopes such as lutetium 177 (177Lu) directly to cancer cells.

The field of theranostics has rapidly evolved and was most recently incorporated into the treatment paradigm for mCRPC. Although prostate-specific membrane antigen (PSMA) positron emission tomography/computed tomography (PET/CT) remains a valuable diagnostic imaging modality, the PSMA tracer also allows for targeted radioligand therapy. PSMA is a transmembrane glutamate carboxypeptidase that is overexpressed in PC cells [27]. Lutetium-17-PSMA therapy involves targeted radionuclide radiotherapy that penetrates the cell membrane by binding to PSMA-positive PC cells. This allows for targeted radiation delivery to PC cells while sparing normal tissue types, which translates to better efficacy and lower toxicity [5, 12••].

Two large trials, TheraP and VISION, recruited men with heavily pretreated mCRPC. Both trials were positive, having met their primary endpoints. VISION recruited men who had previously received ARSIs and up to two lines of taxane based chemotherapy. They were required to have PSMA-positive disease. This was defined as one PSMA-positive metastatic lesion and no PSMA-negative lesions on gallium-68Ga-PSMA-11 PET-CT. Standard therapy in the control arm was physician’s choice but could not include cytotoxic chemotherapy, immunotherapy or PARPi combination. Both rPFS and OS were primary endpoints; accordingly, the study would be positive if either or both endpoints were met. Notably, 61.8% of the trial’s control arm was cabazitaxel naïve, which would have been the next line of therapy, as per the 2019 CARD trial. The VISION trial did not allow for protocol amendments, therefore, the control arm was not allowed to receive cabazitaxel. It was at the physician’s discretion to discontinue experimental treatment if they felt that chemotherapy would be more appropriate [52, 52, 53•].

The TheraP phase II study recruited patients who would have been candidates for cabazitaxel as their next line of treatment, in line with standard practice. Patients with mCRPC were randomized to the Lu-PSMA-617 group or the cabazitaxel group. The primary endpoint was a 50% PSA reduction (PSA50), with secondary endpoints including PFS. The PSA50 response was 66% and 37% in the experimental and control arms with an ORR of 49% and 24%. The median PFS times were 8.7 and 5.1 months, respectively [52].

Generally, Lu-PSMA-617 is well tolerated, with common treatment-related adverse events that include fatigue, nausea, dry mouth, dry eyes, anaemia, thrombocytopenia, leukopenia and diarrhoea. Most of these symptoms are low grade (1–2) and reversible [53•]. A post hoc analysis of the VISION trial examined health-related QoL and pain outcomes. Time to worsening was delayed in the Lu-PSMA group versus the control group in both the functional assessment of cancer therapy–prostate (FACT-P) score and brief pain inventory short form (BPI-SF) pain score. The FACT-P total score was 9.7 vs 2.4 months, HR 0.46, *p* < 0.001, favouring the experimental arm. There was a delay in time to worsening of 7.3 months. The BPI-SF also favoured the experimental arm at 14.3 vs 2.9 months, HR 0.52 *p* < 0.001. The median time to first symptomatic skeletal events (SSEs) or death was longer in the experimental arm at 11.5 months and 6.8 months in the control arm (HR. 0.50, *p* < 0.001) [54, 55].

More recently, the results from the PSMAfore trial were released as abstract only This study examined the use of lutetium-177 vipivotide tetraxetan treatment in a taxane-naïve setting in patients with mCRPC who had progressed on an ARSI. The comparator arm was the ARSI switch. A crossover was permitted from the comparator arm to receive lutetium treatment on progression. The trial was positive and met its rPFS primary endpoint. At a median follow-up of 88.6 months, the rPFS was 12.0 and 5.6 months, respectively, favouring the experimental arm with an HR of 0.43. There was also an improvement in the QoL score, with a total FACT-P score in the experimental group versus the comparator group (7.5 months vs 4.3 months, HR 0.59 *p* < 0.001), as well as a delay in worsening pain (BPI-SF) of 5.0 vs 3.7 months, HR 0.69 *p* < 0.001 [56•].

## Conclusion

Multiple new therapies have been added to the mCRPC treatment algorithm, which has translated to significantly improved overall survival. The genomic profiling of tumours is paramount to enable the identification of actionable mutations to help guide treatment decisions.

Immunotherapy is not a proven effective treatment in an unselected castrate-resistant population. Future trials should consider more biomarker-guided treatment strategies. PARPi are promising, particularly for treating BRCA-mutant and HRR-deficient tumourss. Several upcoming trials will aim to ascertain the use of these inhibitors in combination with other noncytotoxic treatments and their best place in the prostate cancer treatment paradigm.

Despite the ever-expanding list of treatment options, the aim remains to improve long-term outcomes while better palliating symptoms, managing toxicities and preserving patients’ QoL. The challenge, however, is discovering how best to sequence and combine these treatments to maximize their efficacy.

## Data Availability

No datasets were generated or analysed during the current study.

## Abbreviations

PC: Prostate cancer
mCRPC: Metastatic castrate resistance prostate cancer
PSA: Prostate specific antigen
PSMA: Prostate-specific membrane antigen
rPFS: Radiological progression free survival
OS: Overall survival
HRR: Homologous repair recombinant
ARSI: Androgen-receptor signalling inhibitors
ITT: Intention-to-treat
BRCA: Breast cancer gene
NHA: Novel hormonal agent
PARP: Poly(ADP-ribose) polymerase (PARP)
QoL: Quality of life
ctDNA: Circulating tumour DNA
HR: Hazard ratio
PTEN: Phosphatase and tensin homologue
AR: Androgen receptor
HR: Hazard ratio
PD-L1: *Programmed death-ligand 1*
TMB: Tumour mutational burden

## References

[1] Bray F, Ferlay J, Soerjomataram I, Siegel RL, Torre LA, Jemal A (2018). Global cancer statistics 2018: GLOBOCAN estimates of incidence and mortality worldwide for 36 cancers in 185 countries. CA Cancer J Clin.

[2] Fitzpatrick JM (2008). Management of localized prostate cancer in senior adults: the crucial role of comorbidity. BJU Int.

[3] Henríquez I, Roach M 3rd, Morgan TM, Bossi A, Gómez JA, Abuchaibe O, et al. Current and emerging therapies for metastatic castration-resistant prostate Cancer (mCRPC). Biomedicines. 2021;9(9) 10.3390/biomedicines9091247. 10.3390/biomedicines9091247 PMC8468423 34572433

[4] Anantharaman A, Small EJ (2017). Tackling non-metastatic castration-resistant prostate cancer: special considerations in treatment. Expert Rev Anticancer Ther.

[5] Devlies W, Eckstein M, Cimadamore A, Devos G, Moris L, Van den Broeck T, et al. Clinical Actionability of the genomic landscape of metastatic castration resistant prostate Cancer. Cells. 2020;9(11) 10.3390/cells9112494. 10.3390/cells9112494 PMC7698403 33212909

[6] Barbieri CE, Bangma CH, Bjartell A, Catto JW, Culig Z, Grönberg H (2013). The mutational landscape of prostate cancer. Eur Urol.

[7] Boyd LK, Mao X, Lu YJ (2012). The complexity of prostate cancer: genomic alterations and heterogeneity. Nat Rev Urol.

[8] Rodríguez Antolín A, Martínez-Piñeiro L, Jiménez Romero ME, García Ramos JB, López Bellido D, Muñoz Del Toro J (2019). Prevalence of fatigue and impact on quality of life in castration-resistant prostate cancer patients: the vital study. BMC Urol.

[9] Nussbaum N, George DJ, Abernethy AP, Dolan CM, Oestreicher N, Flanders S (2016). Patient experience in the treatment of metastatic castration-resistant prostate cancer: state of the science. Prostate Cancer Prostatic Dis.

[10] Posdzich P, Darr C, Hilser T, Wahl M, Herrmann K, Hadaschik B, et al. Metastatic prostate Cancer-a review of current treatment options and promising new approaches. Cancers (Basel). 2023;15(2) 10.3390/cancers15020461. 10.3390/cancers15020461 PMC9856730 36672410

[11] Sayegh N, Swami U, Agarwal N (2022). Recent advances in the Management of Metastatic Prostate Cancer. JCO Oncol Pract.

[12.••] Sorrentino C, Di Carlo E. Molecular targeted therapies in metastatic prostate Cancer: recent advances and future challenges. Cancers (Basel). 2023;15(11) 10.3390/cancers15112885. This paper is of major importance. This study provides an excellent and comprehensive summary of recent targeted therapies for prostate cancer and future directions. This highlights the urgent need for prostate cancer therapies. 10.3390/cancers15112885 PMC10251915 37296848

[13] Berthold DR, Pond GR, Soban F, de Wit R, Eisenberger M, Tannock IF (2008). Docetaxel plus prednisone or mitoxantrone plus prednisone for advanced prostate cancer: updated survival in the TAX 327 study. J Clin Oncol.

[14] de Bono JS, Oudard S, Ozguroglu M, Hansen S, Machiels JP, Kocak I (2010). Prednisone plus cabazitaxel or mitoxantrone for metastatic castration-resistant prostate cancer progressing after docetaxel treatment: a randomised open-label trial. Lancet.

[15.••] Sumanasuriya S, De Bono J. Treatment of advanced prostate Cancer-a review of current therapies and future promise. Cold Spring Harb Perspect Med. 2018;8(6) 10.1101/cshperspect.a030635. This paper is of major importance. This study outlines current and future prostate cancer treatment is great depth. It also discusses promising biomarkers and its potential use in clinical practice. 10.1101/cshperspect.a030635 PMC5983161 29101113

[16] Lavaud P, Dumont C, Thibault C, Albiges L, Baciarello G, Colomba E (2020). Next-generation androgen receptor inhibitors in non-metastatic castration-resistant prostate cancer. Ther Adv Med Oncol.

[17] Scher HI, Fizazi K, Saad F, Taplin ME, Sternberg CN, Miller K (2012). Increased survival with enzalutamide in prostate cancer after chemotherapy. N Engl J Med.

[18] de Bono J, Mateo J, Fizazi K, Saad F, Shore N, Sandhu S (2020). Olaparib for metastatic castration-resistant prostate Cancer. N Engl J Med.

[19] Smith MR, Scher HI, Sandhu S, Efstathiou E, Lara PN, Evan YY, George DJ, Chi KN, Saad F, Ståhl O, Olmos D (2022). Niraparib in patients with metastatic castration-resistant prostate cancer and DNA repair gene defects (GALAHAD): a multicentre, open-label, phase 2 trial. Lancet Oncol.

[20] de Bono JS, Marcos EC, Laird DA, Fizazi K, Dorff T, Zhao S, van Oort IM, Gasparro D, Calabrò F, Pignata S, Geczi L (2022). TALAPRO-1: Talazoparib monotherapy in metastatic castration-resistant prostate cancer (mCRPC) with DNA damage response alterations (DDRm)–exploration of tumors genetics associated with prolonged benefit. Ann Oncol.

[21] Beer TM, Armstrong AJ, Rathkopf D, Loriot Y, Sternberg CN, Higano CS (2017). Enzalutamide in men with chemotherapy-naive metastatic castration-resistant prostate cancer: extended analysis of the phase 3 PREVAIL study. Eur Urol.

[22] Mostaghel EA. Abiraterone in the treatment of metastatic castration-resistant prostate cancer. Cancer Manag Res. 2014:39–51. 10.2147/CMAR.S39318 PMC3912049 24501545

[23] Rehman Y, Rosenberg JE (2012). Abiraterone acetate: oral androgen biosynthesis inhibitor for treatment of castration-resistant prostate cancer. Drug Des Devel Ther.

[24] de Bono JS, Logothetis CJ, Molina A, Fizazi K, North S, Chu L (2011). Abiraterone and increased survival in metastatic prostate cancer. N Engl J Med.

[25] Ryan CJ, Smith MR, Fizazi K, Saad F, Mulders PF, Sternberg CN (2015). Abiraterone acetate plus prednisone versus placebo plus prednisone in chemotherapy-naive men with metastatic castration-resistant prostate cancer (COU-AA-302): final overall survival analysis of a randomised, double-blind, placebo-controlled phase 3 study. Lancet Oncol.

[26] Kwan EM, Wyatt AW, Chi KN (2022). Towards clinical implementation of circulating tumors DNA in metastatic prostate cancer: opportunities for integration and pitfalls to interpretation. Front Oncol.

[27] Hatano K, Nonomura N (2022). Genomic profiling of prostate Cancer: an updated review. World J Mens Health.

[28] Nakazawa M, Paller C, Kyprianou N (2017). Mechanisms of therapeutic resistance in prostate Cancer. Curr Oncol Rep.

[29] Hernando Polo S, Moreno Muñoz D, Rosero Rodríguez AC, Silva Ruiz J, Rosero Rodríguez DI, Couñago F. Changing the history of prostate Cancer with new targeted therapies. Biomedicines. 2021;9(4) 10.3390/biomedicines9040392. 10.3390/biomedicines9040392 PMC8067446 33917592

[30] Cannan WJ, Pederson DS (2016). Mechanisms and consequences of double-Strand DNA break formation in chromatin. J Cell Physiol.

[31] Rose M, Burgess JT, O'Byrne K, Richard DJ, Bolderson E (2020). PARP inhibitors: clinical relevance, mechanisms of action and Tumours resistance. Front Cell Dev Biol.

[32] Schaeffer E, Srinivas S, Antonarakis ES, Armstrong AJ, Bekelman JE, Cheng H (2021). NCCN guidelines insights: prostate Cancer, version 1.2021. J Natl Compr Cancer Netw.

[33] Gillessen S, Armstrong A, Attard G, Beer TM, Beltran H, Bjartell A (2022). Management of Patients with advanced prostate Cancer: report from the advanced prostate Cancer consensus conference 2021. Eur Urol.

[34] Mateo J, de Bono JS, Fizazi K, Saad F, Shore N, Sandhu S (2024). Olaparib for the treatment of patients with metastatic castration-resistant prostate Cancer and alterations in BRCA1 and/or BRCA2 in the PROfound trial. J Clin Oncol.

[35] Asim M, Tarish F, Zecchini HI, Sanjiv K, Gelali E, Massie CE (2017). Synthetic lethality between androgen receptor signalling and the PARP pathway in prostate cancer. Nat Commun.

[36] Li L, Karanika S, Yang G, Wang J, Park S, Broom BM, et al. Androgen receptor inhibitor-induced "BRCAness" and PARP inhibition are synthetically lethal for castration-resistant prostate cancer. Sci Signal. 2017;10(480) 10.1126/scisignal.aam7479. 10.1126/scisignal.aam7479 PMC5855082 28536297

[37] Saad F, Clarke NW, Oya M, Shore N, Procopio G, Guedes JD (2023). Olaparib plus abiraterone versus placebo plus abiraterone in metastatic castration-resistant prostate cancer (PROpel): final prespecified overall survival results of a randomised, double-blind, phase 3 trial. Lancet Oncol.

[38] Chi KN, Sandhu S, Smith MR, Attard G, Saad M, Olmos D (2023). Niraparib plus abiraterone acetate with prednisone in patients with metastatic castration-resistant prostate cancer and homologous recombination repair gene alterations: second interim analysis of the randomized phase III MAGNITUDE trial. Ann Oncol.

[39] Agarwal N, Azad AA, Carles J, Fay AP, Matsubara N, Heinrich D (2023). Talazoparib plus enzalutamide in men with first-line metastatic castration-resistant prostate cancer (TALAPRO-2): a randomised, placebo-controlled, phase 3 trial. Lancet.

[40] Gasmi A, Roubaud G, Dariane C, Barret E, Beauval JB, Brureau L, et al. Overview of the development and use of Akt inhibitors in prostate Cancer. J Clin Med. 2021;11(1) 10.3390/jcm11010160. 10.3390/jcm11010160 PMC8745410 35011901

[41] Lotan TL, Heumann A, Rico SD, Hicks J, Lecksell K, Koop C (2017). PTEN loss detection in prostate cancer: comparison of PTEN immunohistochemistry and PTEN FISH in a large retrospective prostatectomy cohort. Oncotarget.

[42] Jamaspishvili T, Berman DM, Ross AE, Scher HI, De Marzo AM, Squire JA (2018). Clinical implications of PTEN loss in prostate cancer. Nat Rev Urol.

[43] Crabb SJ, Griffiths G, Marwood E, Dunkley D, Downs N, Martin K (2021). Pan-AKT inhibitor Capivasertib with docetaxel and prednisolone in metastatic castration-resistant prostate Cancer: a randomized, placebo-controlled phase II trial (ProCAID). J Clin Oncol.

[44] Crabb SJ, Ye D-W, Uemura H, Morris T, Gresty C, Logan J (2023). CAPItello-280: a phase III study of capivasertib and docetaxel versus placebo and docetaxel in metastatic castration-resistant prostate cancer.

[45] Sweeney C, Bracarda S, Sternberg CN, Chi KN, Olmos D, Sandhu S (2021). Ipatasertib plus abiraterone and prednisolone in metastatic castration-resistant prostate cancer (IPATential150): a multicentre, randomised, double-blind, phase 3 trial. Lancet.

[46] Shorning BY, Dass MS, Smalley MJ, Pearson HB (2020). The PI3K-AKT-mTOR pathway and prostate cancer: at the crossroads of AR, MAPK, and WNT signaling. Int J Mol Sci.

[47] Graff JN, Liang LW, Kim J, Stenzl A (2021). KEYNOTE-641: a phase III study of pembrolizumab plus enzalutamide for metastatic castration-resistant prostate cancer. Future Oncol.

[48] Gratzke C, Kwiatkowski M, De Giorgi U, Martins da Trindade K, De Santis M, Armstrong AJ (2022). KEYNOTE-991: pembrolizumab plus enzalutamide and androgen deprivation for metastatic hormone-sensitive prostate cancer. Future Oncol.

[49] Antonarakis ES, Park SH, Goh JC, Shin SJ, Lee JL, Mehra N (2023). Pembrolizumab plus Olaparib for patients with previously treated and biomarker-unselected metastatic castration-resistant prostate Cancer: the randomized, open-label, phase III KEYLYNK-010 trial. J Clin Oncol.

[50] Antonarakis ES, Piulats JM, Gross-Goupil M, Goh J, Ojamaa K, Hoimes CJ (2020). Pembrolizumab for treatment-refractory metastatic castration-resistant prostate Cancer: multicohort, open-label phase II KEYNOTE-199 study. J Clin Oncol.

[51] Handa S, Hans B, Goel S, Bashorun HO, Dovey Z, Tewari A (2020). Immunotherapy in prostate cancer: current state and future perspectives. Ther Adv Urol.

[52] Hofman MS, Emmett L, Violet JY, Zhang A, Lawrence NJ, Stockler M (2019). TheraP: a randomized phase 2 trial of 177Lu-PSMA-617 theranostic treatment vs cabazitaxel in progressive metastatic castration-resistant prostate cancer (clinical trial protocol ANZUP 1603). BJU Int.

[53.•] Morris MJ, De Bono JS, Chi KN, Fizazi K, Herrmann K, Rahbar K, et al. Phase III study of lutetium-177-PSMA-617 in patients with metastatic castration-resistant prostate cancer (VISION). Am Soc Clin Oncol. 2021; This paper is important. This study demonstrated that in this heavily pretreated mCRPC population with a significant cancer burden, lutetium treatment did not result in severe toxicity and improved quality of life. The results were reported using validated patient-reported outcomes

[54] Fizazi K, Herrmann K, Krause BJ, Rahbar K, Chi KN, Morris MJ (2023). Health-related quality of life and pain outcomes with [177Lu] Lu-PSMA-617 plus standard of care versus standard of care in patients with metastatic castration-resistant prostate cancer (VISION): a multicentre, open-label, randomised, phase 3 trial. Lancet Oncol.

[55] Sartor AO, Morris MJ, Messman R, Krause BJ (2020). VISION: an international, prospective, open-label, multicenter, randomized phase III study of 177Lu-PSMA-617 in the treatment of patients with progressive PSMA-positive metastatic castration-resistant prostate cancer (mCRPC).

[56.•] Sartor O, Gauna DC, Herrmann K, de Bono J, Shore N, Chi K (2023). LBA13 phase III trial of [177Lu] Lu-PSMA-617 in taxane-naive patients with metastatic castration-resistant prostate cancer (PSMAfore). Ann Oncol.

